# Closed-Loop, Multichannel Experimentation Using the Open-Source NeuroRighter Electrophysiology Platform

**DOI:** 10.3389/fncir.2012.00098

**Published:** 2013-01-18

**Authors:** Jonathan P. Newman, Riley Zeller-Townson, Ming-Fai Fong, Sharanya Arcot Desai, Robert E. Gross, Steve M. Potter

**Affiliations:** ^1^Laboratory for Neuroengineering, Department of Biomedical Engineering, Georgia Institute of Technology and Emory University School of MedicineAtlanta, GA, USA; ^2^Department of Physiology, Emory University School of MedicineAtlanta, GA, USA; ^3^Department of Neurosurgery, Emory University School of MedicineAtlanta, GA, USA; ^4^Department of Neurology, Emory University School of MedicineAtlanta, GA, USA; ^5^Department of Biomedical Engineering, Georgia Institute of Technology and Emory University School of MedicineAtlanta, GA, USA

**Keywords:** closed-loop, multichannel, real-time, multi-electrode, micro-electrode array, electrophysiology, open-source, network

## Abstract

Single neuron feedback control techniques, such as voltage clamp and dynamic clamp, have enabled numerous advances in our understanding of ion channels, electrochemical signaling, and neural dynamics. Although commercially available multichannel recording and stimulation systems are commonly used for studying neural processing at the network level, they provide little native support for real-time feedback. We developed the open-source NeuroRighter multichannel electrophysiology hardware and software platform for closed-loop multichannel control with a focus on accessibility and low cost. NeuroRighter allows 64 channels of stimulation and recording for around US $10,000, along with the ability to integrate with other software and hardware. Here, we present substantial enhancements to the NeuroRighter platform, including a redesigned desktop application, a new stimulation subsystem allowing arbitrary stimulation patterns, low-latency data servers for accessing data streams, and a new application programming interface (API) for creating closed-loop protocols that can be inserted into NeuroRighter as plugin programs. This greatly simplifies the design of sophisticated real-time experiments without sacrificing the power and speed of a compiled programming language. Here we present a detailed description of NeuroRighter as a stand-alone application, its plugin API, and an extensive set of case studies that highlight the system’s abilities for conducting closed-loop, multichannel interfacing experiments.

## 1 Introduction

Multi-electrode neural interfacing systems, such as planar electrode arrays, silicon probes, and microwire arrays are commonly used to record spatially distributed neural activity *in vitro* and *in vivo*. Advances in nanoscale fabrication techniques have continued to push channel counts and electrode resolution (Du et al., 2011; Fiscella et al., 2012; Robinson et al., 2012), allowing for increasingly detailed measurements of network activity states. Because multi-electrode neural interfaces provide many parallel measurements, they can be used to rapidly estimate ensemble features of network activity (e.g., the population firing rate or network-level synchronization). This makes them well suited for real-time applications.

However, most commercial software interfaces for controlling multichannel hardware lack flexible support for real-time, bi-directional communication with neural tissue. Additionally, commercial software is often hard to integrate into complex multicomponent experimental configurations. As a result, multichannel hardware has not been incorporated into closed-loop interfacing schemes to the degree of single-cell recording systems, such as voltage and dynamic clamp (Cole, 1949; Marmont, 1949; Hamill et al., 1981; Prinz et al., 2004; Arsiero et al., 2007; Kispersky et al., 2011). There are some exceptions to this trend (Jackson et al., 2006b; Azin and Guggenmos, 2011; Zanos et al., 2011). These systems are typically limited to low channel counts and/or low recording resolution in order to achieve embedded real-time processing at the recording site using a microcontroller or DSP. This approach has clear advantages for experiments on freely moving animals, but is limited in terms of input and output bandwidth, processing power to enable complex experimental protocols, and ease of programming. Neuroscience research would benefit from a multichannel acquisition platform that (1) enables bi-directional interaction with neuronal networks, (2) is practical for everyday use, (3) is straightforwardly extensible for complex closed-loop protocols, (4) works with a variety multi-electrode interfaces, (5) provides large channel counts and high recording resolution, and (6) is low cost. This type of system would be particularly applicable to three areas of neuroscience research:

- Feedback Control of Network Variables: Neuronal networks are complex systems with many recurrently interacting components. This often results in ambiguity in cause and effect relationships between network variables (Rich and Wenner, 2007; Turrigiano, 2011). Feedback control can be used to parse variables of neural activation that are causally linked (Cole, 1949). Feedback control of network-level variables (e.g., population firing rate, neuronal synchronization, or neurotransmission levels) can potentially clarify their causal relationships (Wagenaar et al., 2005; Wallach et al., 2011).
- Artificial Embodiment: Dissociated neural cultures, slice preparations, and anesthetized or paralyzed animals allow stable electrophysiological access but cannot engage in natural behaviors with their environment. By artificially embodying reduced neuronal preparations using a virtual environment or a robot, experimental access is maintained while neural tissue is engaged in complex behaviors (Reger et al., 2000; DeMarse et al., 2001; Ahrens et al., 2012).
- Clinical Applications: Responsive (Morrell, 2011) or predictive (Mormann et al., 2007) application of neural therapies have the potential to improve the efficacy and safety of treatments that are currently used in open-loop. Examples include brain stimulation and local drug perfusion techniques that are used to treat movement disorders, clinical depression, chronic pain, and epilepsy. Additionally, electrical stimuli delivered to one region of motor cortex in response to spiking activity in another motor area has been shown to facilitate a functional reorganization of motor output, indicating a potential role for activity-dependent stimulation in rehabilitation therapy (Jackson et al., 2006a).

Here, we present substantial improvements to NeuroRighter, an open-source, multichannel neural interfacing platform which we designed specifically to enable bi-directional, real-time communication with neuronal networks (Rolston et al., 2009a, 2010). In the first half of the paper, we provide a description of NeuroRighter’s capabilities, including an application programming interface (API) that facilitates the creation of custom real-time experiment protocols. In the second half of the paper, we demonstrate these features with a variety of case studies. Each case-study highlights a different aspect of NeuroRighter’s abilities in the areas of network-level feedback control, artificial embodiment, and closed-loop control of aberrant activity states in freely moving animals.

## 2 The NeuroRighter Multichannel Electrophysiology Platform

NeuroRighter is an open-source, low-cost multichannel electrophysiology system designed for bi-directional neural interfacing (Rolston et al., 2009a, 2010). A complete system, including all necessary electronics and a host computer, can be assembled for less than $10,000 USD. The NeuroRighter software is free. Extensive documentation on the construction and usage of a NeuroRighter system is available online^1^. NeuroRighter’s source code, the API reference, and demonstration closed-loop protocol code, are available from the NeuroRighter code repository^2^. Questions on NeuroRighter assembly and usage can be submitted to the NeuroRighter-Users forum^3^. Tutorials on API usage are provided in sections 1 and 2 of the Supplementary Material.

### 2.1 Hardware

Here we provide a summary of NeuroRighter’s hardware building blocks. Hardware components can be used with neural interfaces designed for applications both *in vivo* and *in vitro*. Printed circuit board (PCB) performance specifications are provided in (Rolston et al., 2009a) and layouts are available online. A complete NeuroRighter system meets or exceeds the performance of commercial alternatives in terms of noise levels, stimulation channel count, stimulation recovery times, and flexibility (Rolston et al., 2009a). NeuroRighter’s PCBs are designed to be modular: electrode interfacing and stimulation PCBs have identical footprints and use vertical headers to route power between boards. This allows interfacing PCBs to be stacked on top of one another for increased channel counts and the use of a single DC power supply (or set of batteries) for all hardware.

#### 2.1.1 ADC/DAC boards

NeuroRighter uses National Instruments (NI; National Instruments Corp, Austin, TX, USA) data acquisition hardware driven with NI’s hardware control library, DAQmx. NI PCI-6259, PCIe-6259, PCIe-6353, and PCIe-6363 16-bit, 1 M sample/sec data acquisition cards are currently supported. Each card supports 32 analog inputs (AI), 4 analog outputs (AO), and 48 I/O-configurable digital channels. NI SCB-68 screw-terminal connector boxes are used to interface each data acquisition card with external hardware. Up to 3 cards can be used in a single NeuroRighter system to meet channel count requirements.

#### 2.1.2 Multichannel amplifier interfacing boards

NeuroRighter provides two types of PCB to interface the NI data acquisition cards with multi-electrode amplifier systems. For *in vivo* applications, a 16-channel filter module provides 1.6X signal buffering, anti-aliasing filtering (−3 dB point at 8.8 KHz), DC offset subtraction (−3 dB point at 1 Hz), and regulated power to the headstage. Up to four of these modules can be stacked together in order to meet channel count requirements. For *in vitro* applications, a 68 channel conversion board provides power and signal routing for planar electrode array amplifier systems, e.g., Multichannel Systems’ 60 channel amplifiers (Multichannel Systems, Reutlingen, Germany), which have a manufacturer settable pass-band. Both boards interface with the SCB-68 connector boxes using 34-channel ribbon cables, wired as signal/ground pairs to reduce capacitive crosstalk between adjacent lines during stimulation.

#### 2.1.3 Electrical micro-stimulation hardware

NeuroRighter includes all-channel (up to 64 electrodes) stimulation capabilities for both *in vivo* and *in vitro* systems. This system is based upon the circuits presented in (Wagenaar and Potter, 2004; Wagenaar et al., 2004) and includes two separate PCBs: (1) a voltage- or current-controlled signal generation PCB, and (2) a signal multiplexing and isolation PCB to select different electrodes for stimulation and isolate recording electrodes from stimulation cables between stimulus pulses.

(1) *Signal generation board*. The signal generation PCB is identical for all applications. This board provides both voltage controlled or constant current stimulation modes. It stacks into the amplifier interfacing board(s) and therefore does not require an additional power source. Aside from stimulus generation, this PCB can be used to perform electrode impedance measurements, which are useful for diagnosing the health of micro-electrodes and their insulated leads, and for electroplating (Desai et al., 2010). Only one signal generation PCB is required for up to 64 electrodes.
(2) *Signal multiplexing boards*. Stimulus multiplexing and isolation occurs at PCBs that piggyback directly on electrode pre-amplifiers. These PCBs are located close to the initial stages of electrode amplification so that the recording amplifier can be isolated from long electrical leads, which reduces capacitive pickup. Because recording amplifiers (e.g., headstages *in vivo* or multichannel amplifiers *in vitro*) come in many shapes and sizes, the design of the multiplexer PCBs is application dependent. For *in vivo* applications, we have designed multiplexer systems that use an 18-pin Omnetics Nano connector, which interfaces with headstages from Triangle Biosystems (Durham, NC), Tucker-Davis Technologies (Alachua, FL), and Neurolinc Corporation (New York, NY), among others (Rolston et al., 2009a). This board employs a single 1-of-16 multiplexer. For *in vitro* applications, four separate multiplexing modules, each of which houses two 1-of-8 multiplexers, plug directly into exposed 0.1″ pitch sockets of a 60 channel Multichannel Systems amplifier (Wagenaar and Potter, 2004). The creation of custom multiplexer boards or adapters for other systems is straightforward due to the simplicity of these PCBs (they generally consist of a single multiplexer integrated circuit).

#### 2.1.4 Generic I/O

NeuroRighter provides 4 analog output channels and 32 bits of programmable digital I/O for controlling or recording digital signals from laboratory equipment. An auxiliary set of up to 32 analog input channels and 32 bits of digital I/O can also be used. Channel counts of generic I/O in a NeuroRighter system depend on the number of data acquisition cards in the user’s system, and the amount of analog input channels reserved for the electrodes.

NeuroRighter’s hardware serves as an adaptable interface between multi-electrode sensors and data acquisition cards for recording and microstimulation. There are many other options for routing signals to and from the acquisition cards. Therefore, except for the acquisition cards themselves, the hardware we present here is not required to make use of NeuroRighter’s software.

### 2.2 Software

The NeuroRighter software application was written in C# (pronounced “C-Sharp”). C# is a modern, general purpose, object-oriented programming language. The software is free and its source code is maintained on a publicly accessible repository^4^. For standard installations, NeuroRighter is distributed as an installation package for 32- or 64-bit Windows operating systems (Microsoft Corp., Redmond, WA). NeuroRighter installations contain two software components:

1. A stand-alone multichannel recording and stimulation application. This includes a graphical user interface (GUI) for data visualization, hardware configuration, data filtering, spike detection and sorting, all-channel stimulation, stimulus artifact rejection, and data recording (section 2.2.1).
2. An application programming interface (API) that allows NeuroRighter to be used as a real-time hardware interface and data server for user-coded protocols (section 2.2.2).

#### 2.2.1 The NeuroRighter application

**Figure 1:**
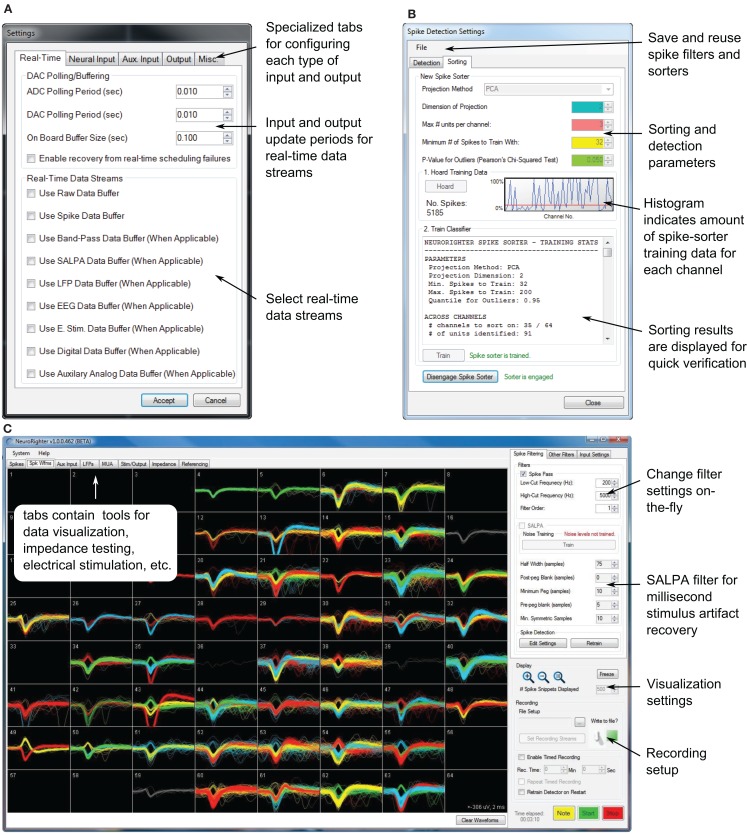
**Portions of NeuroRighter’s graphical user interface**. **(A)** The hardware settings interface. **(B)** The spike-detection filter and spike sorting interface. **(C)** The main application window. Sorted spike waveforms recorded from a 59-channel, planar electrode array are shown on the spike visualization tab of the main GUI. The position of each waveform corresponds to the position of the recording electrode on which it was detected.

As a stand-alone application, NeuroRighter can be used for high-quality multichannel recordings (16-bit resolution, 31 k Samples/sec/channel) and all-channel stimulation protocols. NeuroRighter’s graphical interface is organized into tabbed pages, each of which encapsulates a particular group of functions or visualization tools (Figure 1). In the following section, we discuss the main functional aspects of the stand-alone NeuroRighter application.

##### 2.2.1.1 Main interface

The main NeuroRighter interface (Figure 1C) is an access point for all of the application’s functionality. It facilitates user manipulation of hardware settings, online filter settings, data visualization windows, stimulation tools, and other features, which are discussed below. Additionally, some recording settings can be manipulated within the main interface itself:

***Online acquisition settings***. Many filter settings can be adjusted during data collection. This allows the user to fine tune acquisition settings while gaining visual feedback of the effect on incoming data streams. Bandpass, spike detection, and spike sorting parameters can be adjusted during a recording.

***Data visualization***. Data visualization tools in NeuroRighter use the Microsoft XNA game development framework. This ensures that online visualization does not consume CPU cycles by offloading plotting routines to a supported graphics card. Visualization tools are provided for single-unit activity, local field potentials (LFP), multiunit activity (MUA), electroencephalograph (EEG) traces, and auxiliary analog input streams. Additionally, overlay plots are used to display sorted spike waveforms for each channel (Figure 1C).

***File saving***. Data streams selected by the user are written to disk with a unique file extension that designates their type. These binary files can be read with MATLAB (Mathworks, Natick, MA) functions included with NeuroRighter installations.

##### 2.2.1.2 Hardware configuration

Correctly specifying mixed digital and analog signal routing, clock synchronization, and trigger synchronization on a multi-board data acquisition system can be complicated. NeuroRighter simplifies this process using a graphical hardware settings interface (Figure 1A). Here, the user specifies the types of signals carried by the NI acquisition cards in his or her system, amplifier gain settings, auxiliary input and output channels, options for electrode impedance measurement, signal referencing, and real-time data streaming options. Upon closing the settings dialog, NeuroRighter performs the required signal routing and clock synchronization. All NI cards are synchronized to a single clock oscillator using an NI real-time system integration bus (RTSI, Figure 3).

##### 2.2.1.3 Time-series filtering

Incoming data from the A/D converters are passed through a cascade of digital filters to produce different neural data streams. First, channel voltages are passed through several linear filters to extract frequency bands for single-unit activity (≃200–5000 Hz) and LFP (≃1–500 Hz). MUA, which reflects the firing rate of neurons within the vicinity of the recording electrode, is extracted by rectifying and then low pass filtering the single-unit activity data stream (Supèr and Roelfsema, 2005).

In addition to traditional filtering methods, NeuroRighter provides several specialized filtering options. Common-mode noise sources such as AC mains pickup or movement artifacts in freely moving animals can corrupt neural recordings. NeuroRighter allows the mean or median of all recording electrodes (with appropriate scaling) to be subtracted from individual electrode voltage streams to combat common-mode interference (Rolston et al., 2009b). This is an effective method for reducing non-periodic common-mode interference, such as movement artifacts, where template subtraction methods are inappropriate. Finally, NeuroRighter includes an implementation of the SALPA filter (Wagenaar and Potter, 2002), which subtracts locally fit cubic splines from electrode traces following the application of a stimulus pulse. This removes the capacitive artifacts from non-saturated recording channels and allows online action potential detection within 2 ms after a stimulus pulse.

Sampling rates for different data streams can be set independently. Filter settings (pass-band and filter order) can be modified during data acquisition (Figure 1C). Raw data, as well as the result of each filtering stage, yield separate data streams (Table 1).

**Table 1:** **Overview of NeuroRighter’s input and output streams**.

Input	Source	Server (DataSrv)	Buffer type	Max. channel count
	Raw electrodes	RawElectrodeSrv	Circular double[][]	64
	SALPA Filter	SalpaSrv	Circular double[][]	64
	Spike-band filter	SpikeBandSrv	Circular double[][]	64
	Spike filter	SpikeSrv	List <SpikeEvent>	64 or No. units
	LFP filter	LFPSrv	Circular double[][]	64
	EEG filter	EEGSrv	Circular double[][]	64
	MUA filter	MUASrv	Circular double[][]	64
	Electrical stimuli	ElecStimuliSrv	List <SpikeEvent>	64
	Auxiliary analog	AuxAnalogSrv	Circular double[][]	32
	Auxiliary digital	AuxDigitalSrv	List <DigitalEvent>	32 bits

**Output**	**Source**	**Server (StimSrv)**	**Buffer type**	**Max. channel count**

	Electrical stimuli	StimOut	List <StimulusEvent>	64
	Analog output	AnalogOut	List <AnalogEvent>	4
	Digital output	DigitalOut	List <DigitalEvent>	32 bits

*Each stream is accessed using a dedicated server that includes functions for reading from, or writing to, its data buffer*.

##### 2.2.1.4 Spike filtering

Spike filtering in NeuroRighter is a three-step process: (1) detection, (2) validation, and (3) sorting. NeuroRighter detects spikes using a threshold criterion that compares individual voltage samples to the estimated RMS voltage on the corresponding electrode. Upon threshold crossing, a peak-aligned voltage “snippet” is extracted from the raw voltage stream. Each snippet is validated using a series of *ad hoc* criteria based upon waveform slope, width, and peak-to-peak amplitude. Finally, spikes can be sorted online using an automated Gaussian mixture modeling algorithm. Details of the spike detection and sorting algorithms used by NeuroRighter are provided in section 3 in the Supplementary Material.

The spike detection/sorting configuration is controlled through a child GUI (Figure 1B). All relevant spike detection, validation, and sorting parameters are under user control and are manipulated using the spike detection GUI. Because spike-detection settings are changed using a secondary GUI, the effects of parameter changes can be simultaneously monitored on the visualization tabs in the main interface while data collection occurs. A complete list of these parameters is shown in Table S1 in the Supplementary Material. Spike filters, including trained spike sorters, can be saved and reused.

##### 2.2.1.5 Stimulation

NeuroRighter provides several options for delivering complex stimulus patterns to neural tissue either manually through the NeuroRighter application or using scripted protocols. Simple, periodic stimulation protocols, consisting of single or double phase, square, current- or voltage-controlled pulses on any electrode, can be performed directly from the main GUI. Stimuli can be triggered “on demand” in response to a mouse click or by using hardware-timed, periodic sequence of triggers.

**Figure 2:**
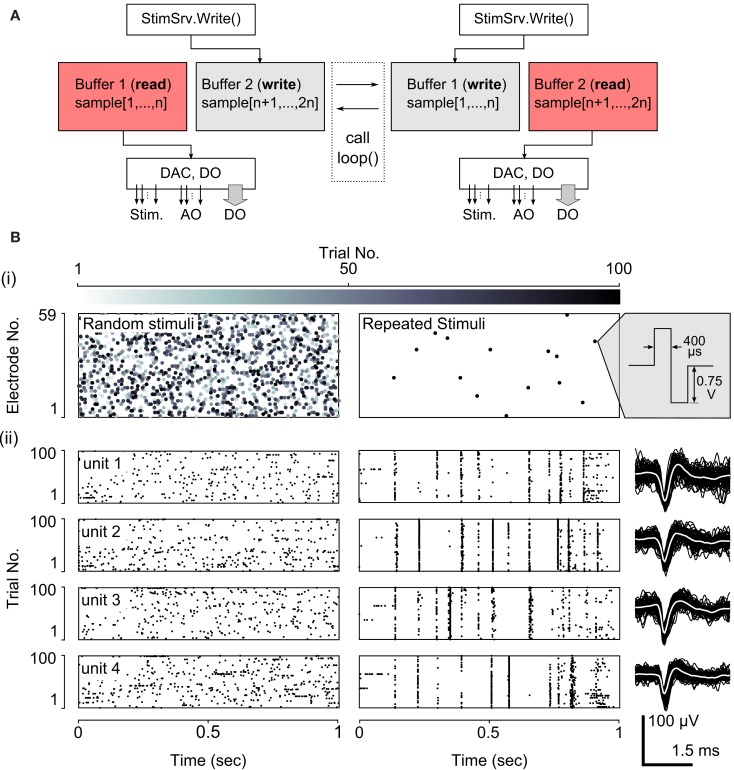
**NeuroRighter’s StimSrv subsystem**. **(A)** To deliver complex, non-periodic stimuli, NeuroRighter uses a double-buffering system. This allows samples to be generated and written to the NI cards’ analog and digital outputs simultaneously. At a given instant, one buffer is reserved for reading (pink) and one from writing (gray). When the all samples in the read buffer are generated, the buffers switch roles, allowing seamless delivery of constantly varying stimulus patterns and generic analog and digital signals. When using StimSrv for closed-loop protocols, the loop() function is called at the instant of a buffer switch. **(B)** Example open-loop stimulus protocol using StimSrv. (i) 100, 1 s Poisson sequences of electrical stimuli (left) and a single repeated Poisson sequence (right), were delivered to a dissociated cortical network (biphasic, voltage controlled, ±0.75V, 800 μs period). Stimulus rasters are shown using a gray-scale to indicate the trial number. For repeated stimuli, stimulus points are overlaid since stimulus delivery is clock-synchronized with the acquisition subsystem. (ii) Rastergrams of 4 units are shown below each stimulus raster, across trials. Example waveforms for each of the 4 units are shown to the right.

Scripted protocols can be used to deliver complex, potentially non-periodic stimulus patterns and to access general purpose analog and digital output lines. Neurorighter uses a double-buffered output engine, called StimSrv (Table 2), to produce arbitrary, hardware-timed stimulation, analog-output, or digital output signals (Table 1, bottom). StimSrv can be accessed on-the-fly using NeuroRighter’s API (section 2.2.2) or with user-written scripts. The schematic in Figure 2A demonstrates how StimSrv delivers uninterrupted output. First, a block of the NI cards’ memory is reserved and divided into two sections, each of which comprises a single output buffer. At a given instant, one buffer is reserved for sample generation and one is available for writing. When the all samples in the read buffer are exhausted, the buffers switch roles, allowing seamless delivery of constantly varying output signals. This allows the delivery of complex, aperiodic stimulation patterns and the orchestration of experimental apparatuses using analog and digital output lines. All output is clock-synchronized to input data streams, allowing *a priori* specification of stimulus delivery times, relative to the start of the experiment, with single-sample precision. Stimulation scripts can be created with a set of MATLAB functions that are included with NeuroRighter installations (see section 1 in the Supplementary Material).

**Table 2:** **Packages included with NeuroRighter’s Plugin API**.

Package	Component	Description
Server	DataSrv	Contains input server objects (Table 1, top)
	StimSrv	Contains output server objects (Table 1, bottom)
Datatypes	MultiChannel Buffer	Circular buffer for time series data
	SpikeEvent	Spike event type (time, channel, waveform, unit)
	DigitalEvent	Digital event type (time, 32-bit port state)
	StimulusEvent	Stimulus event type (time, channel, waveform)
	AuxEvent	Auxiliary voltage event (time, channel, voltage)
NeuroRighter Task	NRTask	Abstract class for real-time NeuroRighter interfacing
Log	Logger	Used for debugging real-time protocols

Figure 2B demonstrates the use of a scripted stimulation protocol to deliver spatio-temporal patterns of electrical stimuli. One-second trials of spatially uniform, and temporally Poisson random stimulus pulses were delivered to a dissociated cortical network. Each trial consisted of either a new, random stimulus realization or a single repeated realization. Each type of stimulus sequence was interleaved with no delay between adjacent trials. Figure 2Bi shows stimulus raster plots for 100 trials each stimulus type, with a gray-scale indicating the stimulus trial. For repeated stimuli, individual trials cannot be seen since the recording and stimulation subsystems are clock-synchronized and every repeated stimulus sequence occupies the same set of samples relative to the start of a trial. Figure 2Bii shows spiking patterns in response to random and repeated stimuli for 4 units across trials. The delivery of repeated stimuli to the network results in extremely reproducible spiking patterns, and non-repeated, random stimuli probe the variability of population spiking response. This type of stimulus protocol is commonly used to estimate the mutual information between a stimulation process and the population spiking response (Strong et al., 1998; Yu et al., 2010).

#### 2.2.2 NeuroRighter’s application programming interface

NeuroRighter installations include an API that facilitates the creation of real-time protocols. The API comprises a set of tools for interacting with NeuroRighter’s input and output streams. Protocols written using the API are externally compiled libraries that can “plug in” to the NeuroRighter application in order to impart real-time and closed-loop functionality. The software packages included with the API are shown in Table 2. Each package contains different set of tools for interacting with NeuroRighter’s data streams. Here we discuss the contents and usage of each of these tools. Additionally, a detailed API reference is available online^5^.

##### 2.2.2.1 NeuroRighterTask

User-defined protocols employ the NeuroRighter application as a real-time data server. These protocols are inherited from a base component called NRTask, which belongs to the NeuroRighterTask package. Closed-loop protocols created with the plugin API are derived from NRTask (see section 2 in the Supplementary Material for details). Three functions included in NRTask can then be accessed to impart real-time functionality.

1. NRTask.Setup(): This function is called when the base NRTask component is instantiated. It allows one-time setup operations to take place, such as the declaration of variables, allocation of internal buffers, file streaming setup, GUI initialization, etc.
2. NRTask.Loop(): This function is executed periodically by a hardware-timed clock. Execution periods of 1 to 150 ms are allowed and can be set from the Hardware Settings GUI in the main application (Figure 1A). To achieve closed-loop functionality, code within the Loop function should access other components of the API, most importantly components from the Server and DataTypes packages (Table 2). These packages provide access to incoming neural data streams and output buffers and can be used to form a bi-directional interface with neural tissue. Output can be sent from within the Loop function using the StimSrv package (Table 2) or through natively supported communication interfaces such as TCP/IP ports, serial ports, or USB communication.
3. NRTask.Cleanup(): This function is called a single time when the protocol is stopped from the NeuroRighter GUI. It allows the deconstruction of GUIs, the closure of file streams that may have been created during the execution of the plugin, and other cleanup routines.

**Figure 3:**
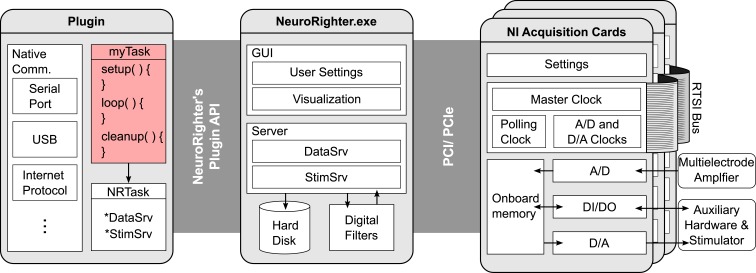
**Conceptual schematic of NeuroRighter’s hardware and software elements**. NeuroRighter serves as a high-level interface between hardware and custom user-written protocols (pink box). NeuroRighter simplifies hardware level programming by using datatypes and methods that are specialized for multichannel neural recording and stimulation. This facilitates the creation of low-latency, closed-loop protocols. Neural signals and secondary data streams are fed into the NI cards’ analog and digital inputs where they are digitalized and stored temporarily in on-board memory. NeuroRighter periodically transfers data from the acquisition cards’ FIFO memory to RAM using direct memory access. Data is then pushed to NeuroRighter’s DataSrv server object. DataSrv serves data to NeuroRighter’s visualization tools, filtering algorithms, and externally compiled plugins. The plugin API provides functions for safe interaction with DataSrv so that custom operations can be performed on incoming data streams. User-written plugins can interact with any of the computer’s native communication ports, or write data back to StimSrv in order to control external hardware as a function of recorded neural signals.

Listing 1A and 1B provide pseudocode for a two real-time plugins that both respond to a spike produced by a particular detected unit. A real-time protocol written using the API will follow the structure of one of these code skeletons, regardless of its complexity. First, the user references the required packages from the API. Next, the plugin is designated to be a child of NRTask, which provides the protocol with automatic access to NeuroRighter’s data servers. Finally, the Setup(), Loop(), and Cleanup() functions are overridden (Listing 1A), or a NewData event is subscribed to (Listing 1B), to impart real-time functionality. After it is compiled (either using Visual Studio or Mono^6^), the plugin can be executed through NeuroRighter’s GUI. Plugin protocols executed through NeuroRighter operate on a high-priority thread to decrease closed-loop response latency. The diagram shown in Figure 3 shows the interaction between a plugin created using the API, the NeuroRighter executable, and hardware. Functional examples of plugin protocols are provided in section 5 of the Supplementary Material.

**Listing 1:**
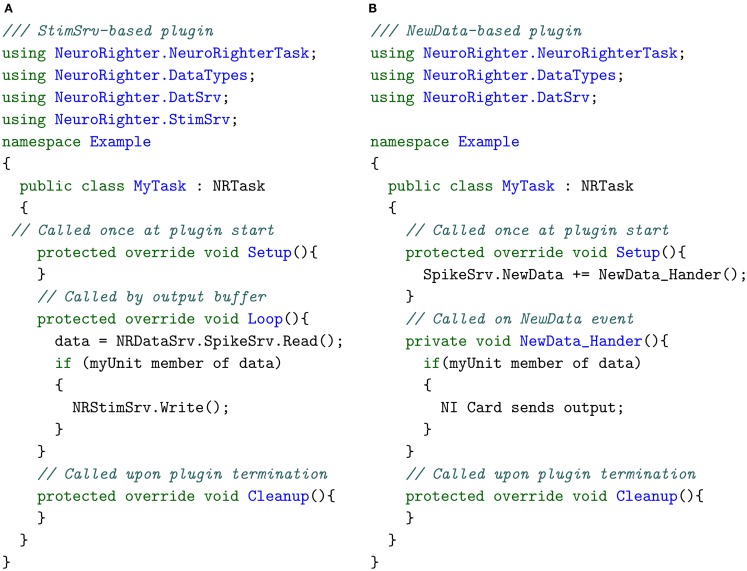
**Code structure for two types of real-time plugin implemented with the API**. **(A)** Pseudocode for a StimSrv-based real-time plugin. **(B)** Pseudocode for real-time plugin triggered by NewData events.

##### 2.2.2.2 Server

Components derived from NRTask have automatic access to NeuroRighter’s input and output servers, which belong to the Server package. There are two banks of data servers: (1) DataSrv, which can be used to read NeuroRighter’s input streams (Table 1, top) and (2) StimSrv, which can be used to write to output streams (Table 1, bottom). DataSrv and StimSrv objects encapsulate isolated data servers, each of which handles a particular data stream. Each server includes methods for reading the hardware clock, reading from and writing to its own data buffer, and accessing stream metadata. Because input and output servers are simultaneously accessible from within a user-defined NRTask, sending output signals (e.g., stimuli) contingent on recorded input is straightforward. The user can select which data streams are sent to DataSrv or available for writing on StimSrv using the Hardware Settings GUI (Figure 1A).

A final important feature of each data server within DataSrv is a NewData event. A NewData event is fired for a given stream each time it receives new data for the A/D card or a digital filter. Functions within a plugin can subscribe to these events so that feedback processing only occurs when new data is acquired. This reduces computational overhead and the latency of the closed-loop response. Plugins that use NewData events to generate feedback are not required to include a Loop() function or to use StimSrv to send output signals. Instead, standard calls to the National Instrument driver library (DAQmx) can be used to access the NI cards’ directly. Alternatively, output can be generated using natively supported external communication protocols (USB, TCP/IP, UDP, serial, etc.). **Listing II. B.2(b)** provides pseudocode for a real-time protocol analogous to **Listing II.B.2(a)**, but using the NewData event to trigger a response. This type of plugin provides a lower response latencies but is less capable of producing complex, precisely timed output signals. A functional example of a NewData-based plugin is provided in section 5.2 in the Supplementary Material.

##### 2.2.2.3 Datatypes

NeuroRighter’s input and output servers operate on high-level data types that encapsulate different forms of multichannel input and output data. These include multichannel buffers for continuous data streams (such as raw electrode voltages or LFP recordings) and discrete event types (such a detected spikes or stimulation events). Extensive documentation on each of these data types is provided in the API reference.

##### 2.2.2.4 Log

The Log package provides accesses to a data logging tool that operates within the NeuroRighter executable, but can be invoked from a user protocol. This tool can be used to write information to a log file using a separate, low-priority thread. This is useful in the development of real-time protocols because core NeuroRighter operations (such as the timing of hardware reads, writes, and other triggers) are logged to this file as well, providing context for messages written from the plugin.

## 3 Case Studies

NeuroRighter’s abilities for orchestrating closed-loop experiments are best demonstrated through example. Here we present five case studies in which protocols created with the API were used to measure NeuroRighter’s closed-loop reaction-time, clamp network firing levels in dissociated cultured cortical networks, react to seizures in freely moving animals with multi-electrode electrical stimulation, and control robots serving as artificial embodiments. Experimental methods, and plugin examples are provided in the section 4 in the Supplementary Material. The plugin code used in these case studies is available for download on NeuroRighter’s code repository. ^7^. Additionally, we provide all code used in the reaction-time case study in section 5 in the Supplementary Material.

### 3.1 Low-latency control of real-time hardware

**Figure 4:**
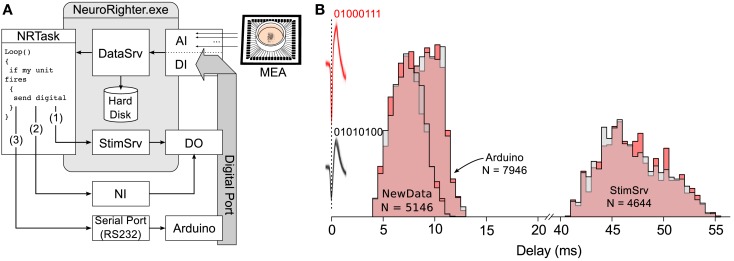
**Estimated loop times for bi-directional communication using different hardware configurations**. **(A)** Schematic of experiment used to test reaction delays for different real-time hardware options. Spikes detected and sorted from 59-channel planar electrode array were passed to the real-time plugin. The plugin determined if a spike originated from one of two units of interest. In the case that a spike was produced by one of the two units, the plugin triggered the generation of a digital word encoding the detected unit using either StimSrv, unbuffered digital output triggered by a NewData event, or an Arduino board. Digital signals were then, recorded though NeuroRighter’s digital input port. **(B)** Normalized histogram of time delays from spikes produced by the two units of interest (action potential waveforms are shown in pink and gray and occur at 0 ms) to the recorded digital signals produced by the plugin to encode the units (01000111 or 01010100). Delay histograms are shown for each unit (pink and gray) and the three different hardware options. N is the number of spikes recorded for each hardware option.

Rapid response times are critical for maintaining a tight feedback loop in which features of incoming data streams (e.g., spikes, EEG, temperature, or animal motion) are used to trigger or adjust the delivery of stimuli. To benchmark the response speed of protocols written using the API, we wrote a protocol that generated output signals in response to recorded action potentials. We picked two sorted units from a dissociated neural culture to serve as triggers for hardware activation. When one of these units fired, it triggered the output of a digital word encoding the identity of the detect unit. These signals serve as a generic stand-in for a stimulation pattern or any other hardware control signal that might be used in a feedback control scheme. Output signals were then recorded using NeuroRighter’s digital input port. The delay between action potential detection and signal generation could then be measured using the same sample clock. A diagram of the experimental protocol is shown in Figure 4A. We wrote protocols to test three hardware options for generating the required digital output:

1. StimSrv: Buffered manipulation of the NI cards using NeuroRighter’s native stimulation server (Figure 2 and **Listing II.B.2(a)**).
2. NewData: Unbuffered manipulation of the NI cards whenever new data enters NeuroRighter’s spike server (**Listing II.B.2(b)**).
3. Arduino: An Arduino ATmega2560-based microcontroller board^8^ communicating via serial port (RS-232).

The response latency, calculated from the time of an action potential peak to the corresponding change in the digital port was calculated for each hardware option (Figure 4). Mean response latencies were 46.9 ± 3.1 ms for rb  StimSrv, 7.1 ± 1.5 ms for NewData, and 9.2 ± 1.3 ms for the Arduino board. Latencies where measured while NeuroRighter performed bandpass filtering, spike detection, spike sorting, data streaming, and data saving for 64 electrode inputs, each sampled at 25 kHz. Experiments were conducted on a desktop computer using an Intel Core i7 processor (Santa Clara, California, USA.) and running running 64-bit Windows Vista.

The differences in reaction latency for different hardware options are a result of both the method used to communicate with the hardware and the how the input sent from NeuroRighter is interpreted and transformed into a physical output signal. The differences in response times for NewData and Arduino are largely attributable to the different communication protocols and command interpretation by the client device. For instance the Arduino used a RS-232 serial interface where as NewData communicates with the NI cards via PCIe. StimSrv’s long latency in comparison to other options is a result of its double buffering system, which requires a relatively long time period between updates to the NI D/A’s output buffer. While StimSrv is slow in comparison to the NewData and microcontroller options, it provides an interface that is easier to use and allows the uninterrupted delivery of arbitrary complex singal outputs. On the other hand, the Arduino and NewData methods can only respond by generating finite-sample or periodic control signals. We have found that StimSrv is fast enough for most of our closed-loop requirements. For this reason, we used StimSrv to generate physical outputs for the remainder of the case studies. However, as demonstrated above, the API’s modularity allows the use of faster hardware options with little change in coding complexity.

### 3.2 Multichannel population firing clamp

The population firing rate is a building block of the neural code. The ability to precisely control population firing in the face of experimental perturbations can be used to understand its role in network function. To demonstrate NeuroRighter’s ability to control the network firing rate, we implemented the feedback controller presented in Wagenaar et al. (2005) to control the firing activity in dissociated cortical cultures grown on 59-channel micro-electrode arrays. This algorithm adjusts the stimulation amplitude of voltage controlled, biphasic pulses on 10 electrodes to desynchronize population firing and force the network firing rate to track target values. The control law is given by

(1) $$v_{k}\left[t+\Delta T\right]=v_{k}\left[t\right]-\alpha v_{k}\left[t\right]\left(\frac{\left\langle f_{u}\left[t\right]\right\rangle }{f^{*}}-1\right),$$

where *v_k_* is the stimulation voltage on electrode *k*, 〈*f_u_*[*t*]〉 is the average firing rate across sorted units detected with the 59 electrode array extending over a 2 s window into the past, *f* * is the target firing rate, Δ*T* is the update period of the feedback loop (as defined within NeuroRighter’s Hardware Settings GUI), and *α* defines the time constant of the feedback controller as

(2) $$\tau_{FB}=\Delta T∕\alpha .$$

We used Δ*T* = 10 ms and α = 0.002 so that *τ_FB_* = 5 s. Electrodes were stimulated at a 10 Hz aggregate frequency (1 Hz per electrode for 10 electrodes) in a random, repeating sequence. Additionally, individual electrode voltages were multiplied by a tuning factor that was inversely proportional to the number of spikes that occurred within 30 ms following a stimulus pulse on that electrode, as described in Wagenaar et al. (2005). This factor equalizes each electrode’s ability to evoke a spiking response, and is critical for achieving the desynchronizing effect of the controller on population activity.

**Figure 5:**
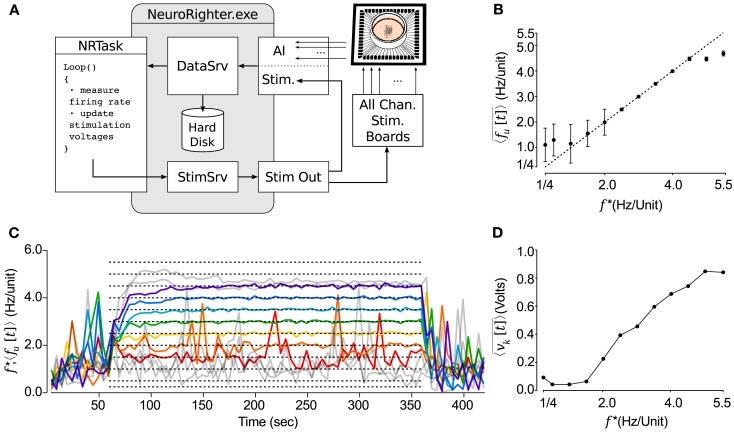
**NeuroRighter can be used to clamp population firing rates *in vitro* using closed-loop electrical stimulation**. **(A)** Schematic of the multi-electrode population firing clamp. **(B)** Step tracking performance is shown for a range of target firing rates, *f* * (dotted lines). The average neuronal firing rate across detected units,〈*f_u_*[*t*]〉 (colored lines), is shown for each step in *f* *. Tracking failures are colored gray. **(C)** Time averaged neuronal firing rate for the last 2.5 min of each 5 min protocol compared to the reference signal, *f* *. The dotted line is identity. **(D)** The mean control voltage across the stimulating electrodes over the final 2.5 min of each step protocol at different values of *f* *.

We used the controller to clamp network firing at target rates for 5 min epochs. These results are shown in Figure 5. The controller was able to achieve target rates within the range of *f* * = 1.5–4.5 Hz/Unit. An animation of neural activity before and during firing-rate clamping is provided in the Supplementary Material.

**Figure 6:**
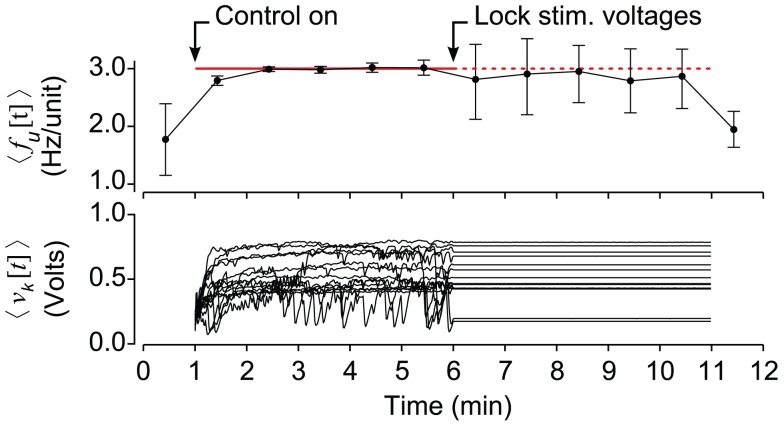
**Closed-loop stimulation is required to robustly clamp population firing**. (Top) The average neuronal firing rate over 1 min periods across 15 trials. Half-way through a multichannel population clamp protocol, real-time voltage updates stop and microstimulation is applied in open-loop. Error bars are ± standard deviation. (Bottom) The mean electrode stimulation voltage across 10 stimulating electrodes, for each of the 15 trials.

The monotonically increasing relationship between the mean stimulation voltage $\bar{\langle v_{k}[t]\rangle }$, and target firing rate *f* * (Figure 5D) might indicate that knowledge of the stimulation voltage versus firing rate relationship is sufficient to design an open-loop controller capable of holding network firing rates. To test this, we clamped firing at *f* * = 3.0 Hz/Unit over 10 min epochs for 15 trials. Five minutes into each 10 min protocol, we stopped updating stimulation voltages on the ten stimulating electrodes, but continued multi-electrode stimulation in open-loop mode (Figure 6). Although the desired mean firing rate was achieved fairly consistently, the open-loop control scheme could not react to the rapid changes in excitability that are typical of cultured cortical networks (Wagenaar et al., 2006b). This variability is reflected in the large range of control signals required to track the target rate over the first 5 min of each trial. As a result the RMS error of 〈*f_u_*[*t*]〉 about *f* * increased by a factor of 5.1 for open-loop compared to closed-loop epochs. The variance of firing during open-loop stimulation is comparable to that of spontaneous (non-evoked) firing behavior that was recorded before the controller was switched on (Figure 6, top).

### 3.3 Long-term population firing clamp with synaptic decoupling

#### 3.3.1 Experiment 1

*In vitro* neural preparations allow continuous experimental access to neural tissue over very long time scales (Potter and DeMarse, 2001), and therefore serve as important models for understanding slowly occurring developmental processes (Turrigiano et al., 1998; Minerbi et al., 2009; Gal et al., 2010). To demonstrate that NeuroRighter is capable of stable closed-loop neural interfacing over long time scales, we used the multi-electrode feedback controller used in section 3.2 for 6 h epochs. This protocol started with a 1 h recording of spontaneous activity. Then, the controller was engaged to clamp population firing to *f* * = 3.0 Hz/Unit for 6 h. Following the clamping protocol, spontaneous network activity was recorded for an additional hour.

**Figure 7:**
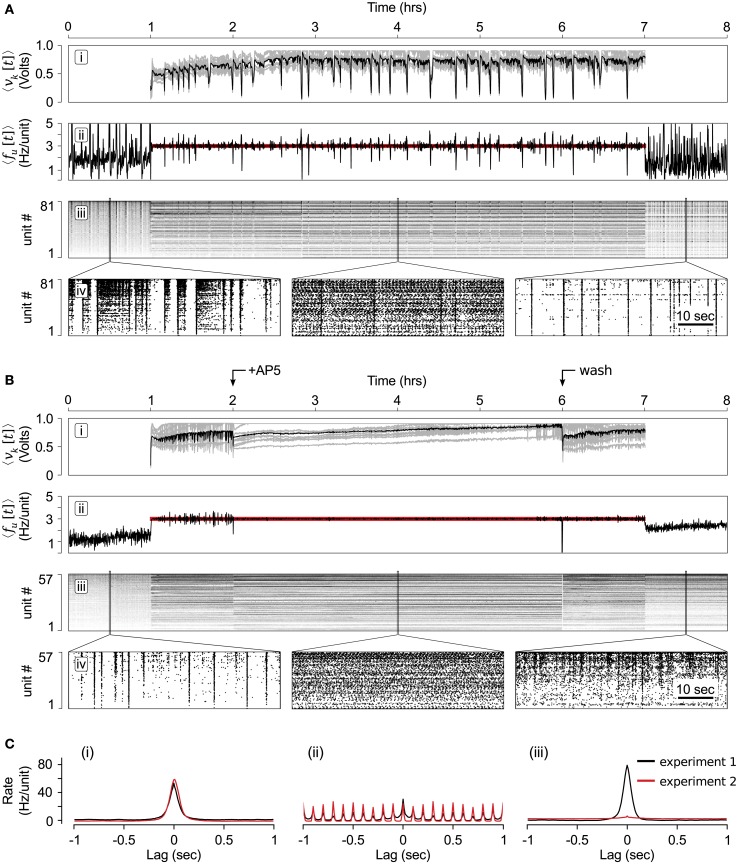
**Long-term population clamp**. **(A)** (i) The mean stimulation voltage (black) and individual electrode stimulation voltages (gray) over the course of the 6-h clamping protocol. (ii) The neuronal firing rate (black) compared to the target rate (red line). (iii) Individual unit firing rates, sorted in order of increasing rate during the 1 h period prior to the start of closed-loop control. (iv) Zoomed rastergrams showing short time scale network spiking before, during and after the controller was engaged. **(B)** Same as **(A)** except that AP5 was added 1 h after the start of the closed-loop controller and removed 4 h later. This is indicated by the arrows at the top of the figure. **(C)** Average pair-wise correlation functions between units for experiments with and without AP5 application (red and black lines, respectively). Cross-correlations were created from spiking data (i) during spontaneous activity before the closed-loop controller was engaged, (ii) half-way through the closed-loop-control period, and (iii) during spontaneous network activity following closed-loop control. The data used to create the correlation functions is centered about locations used to create the rastergrams shown in **(A**iv**)** and **(B**iv**)**. To create the correlation functions, unit firing rates were calculated using 10 ms time bins.

Figure 7A shows the resulting multichannel stimulation signal (Figure 7Ai), neuronal firing rate in relation to *f* * (Figure 7Aii), individual unit firing rates (Figure 7Aiii), and zoomed rastergrams before, during, and after multi-electrode stimulation was applied (Figure 7Aiv). The controller achieved the *f* * = 3.0 Hz/Unit tracking over the duration of the 6 h protocol. Additionally, network activity was desynchronized through most of the control epoch, but occasionally the controller allowed bouts of synchronized network activity (Wagenaar et al., 2006b).

#### 3.3.2 Experiment 2

Spiking and neurotransmission have a strong reciprocal influence on one another, making their individual effects on network development difficult to quantify (Turrigiano, 2011). For instance, *N*-methyl-d-aspartate (NMDA)-ergic neurotransmission plays a large role in sustained network recruitment (Nakanishi and Kukita, 1998). For this reason, long-term changes in the state of *in vitro* networks following the application synaptic blockers (e.g., changes in firing rate, spiking patterns, or synaptic-strength) is difficult to attribute directly to effects on neurotransmission because of secondary, confounding effects on network activity levels. However, the closed-loop population clamp provides a solution to this problem. A firing rate controller has the potential to compensate for changes in network excitability induced by the application of a drug, removing its confounding effect on network activity.

To test this, we used the multichannel population clamp during the bath application of d(−)-2-amino-5-phosphonopentanoic acid (AP5), a competitive antagonist of NMDA receptor. This protocol proceeded identically to experiment 1 except that at 1-h following the start of closed-loop stimulation, NeuroRighter triggered the perfusion of 50 μm AP5 into the culturing medium using a syringe pump and a custom, gas-permeable perfusion lid (Potter and DeMarse, 2001; Figure S5 in the Supplementary Material). Four hours after AP5 was applied, NeuroRighter triggered the pump a second time to perform a series of washes with normal culturing medium that removed AP5 from the bath.

Time-series results of this protocol are shown in Figure 7B. The contents of these plots are analogous to Figure 7A but have arrows to indicate when AP5 was added to, and removed from, the culturing chamber. The controller was able to successfully compensate for changes in network excitability caused by the addition of AP5. Changes in network dynamics were reflected in the control signal, which became smoother in the presence of the AP5 (Figure 7Bi).

#### 3.3.3 Comparing Experiments 1 and 2

Figure 7C shows the average, pair-wise firing rate correlation functions (Tchumatchenko et al., 2010) for 30 randomly selected units from experiment 1 (black lines) and experiment 2 (red lines). Figures 7Ci,iii show the correlation functions of spontaneous network activity before and after the controller was engaged, respectively. Figure 7Cii shows correlation functions for epochs during the clamping phase (which included the AP5 treatment for experiment 2). The periodicity of this correlation function follows the 10 Hz aggregate stimulation frequency during the clamping period.

Intriguingly, although the pair-wise spiking correlations for experiments 1 and 2 were very similar for epochs of spontaneous activity before and during multichannel stimulation (Figures 7Ci,ii), they were remarkably different once the stimulator was turned off (Figure 7Ciii). When AP5 was not present during the clamping phase (experiment 1), the firing correlation between units appeared to be enhanced following multichannel stimulation. In contrast, pair-wise correlations were almost non-existent following the a population clamp in which AP5 was present (experiment 2). Because the firing statistics (firing rate and correlation structure) during the 6-h clamping period were nearly identical for the both experiments 1 and 2, this effect on the correlation structure of network activity can not be due to effects on firing activity, but required blocking NMDAergic transmission. Without the closed-loop controller in place, AP5 would have affected network activity levels, obfuscating the mechanism of AP5’s effect.

This case study demonstrates the ability of the closed-loop controller to quickly adapt to drug-induced changes in network excitability, to decouple network variables that are normally causally intertwined, and to operate robustly over many hours. Additionally, this case study demonstrates NeuroRighter’s ability control peripheral equipment aside from electrical stimulators.

### 3.4 Real-time seizure intervention in freely moving rats

Aside from *in vitro* recording hardware, NeuroRighter can interface with many different types of neural probes, including those designed to record from and stimulate freely moving animals. To demonstrate this, we performed electrical micro-stimulation in response to paroxysmal activity of hippocampal recordings taken from a rat with induced temporal lobe epilepsy. Many studies have shown potentially therapeutic effects of electrical stimulation on epileptic brain tissue, which could serve as an alternative to pharmacological or surgical treatment methods. For instance, electrical stimulation triggered by characteristic field potential abnormalities can potentially abrogate seizures and lead to a decreased frequency of behavioral symptoms (Mormann et al., 2007; Morrell, 2011; Nelson et al., 2011).

**Figure 8:**
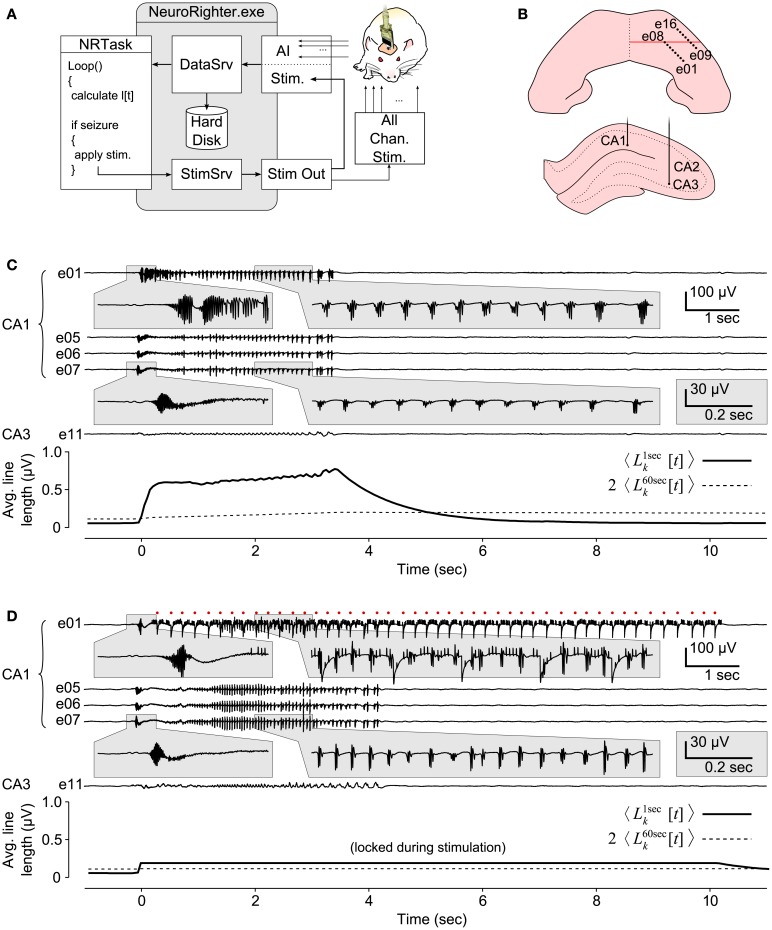
**Closed-loop seizure intervention in a freely moving rat**. **(A)** Schematic of the closed-loop seizure intervention protocol. A 16-channel microwire array, with two rows of 8 electrodes, were used to record LFP signals in the CA1 and CA3 regions of the hippocampus of a epileptic rat. Paroxysmal activity in CA1 triggered the application of multichannel electrical stimulation through the recording electrodes via a stimulation multiplexing board (green). **(B)** Implantation sites of the microwire array. Top view shows the electrode penetration sites (black dots) in the right-dorsal hippocampus. The red line indicates position of the coronal view shown below. **(C)** A 12 s epoch of hippocampal LFPs during a seizure event. Electrodes 1–8 were located in CA1 and 9-16 in CA3. The line length measures, averaged across channels, are shown below the LFP traces. Seizure detection occurs at 0 s. **(D)** Same as **(C)** except with closed-loop stimulation engaged. Electrical stimulation was applied on electrode 1 along with nine other electrodes (not shown). Red dots indicate stimulation times for e01 and stimulation artifacts appear on the LFP trace. e05–e07 and e11 were not used for stimulus application.

We used the plugin API to create a closed-loop protocol that could detect temporal lobe seizures in freely moving rats and react with multi-electrode stimulation (Figure 8A). This control scheme is similar to that of the NeuroPace responsive neurostimulation system (Sun et al., 2008) (NeuroPace Inc., Moutain View, CA, USA), with the exception that we used multi-micro-electrode stimulation instead of driving a single macroelectrode.

Rats were rendered epileptic using focal injections of tetanus toxin into the right-dorsal hippocampus (Hawkins and Mellanby, 1987; see section 4C in the Supplementary Material). LFPs were recorded from CA1 and CA3 regions of the hippocampus using a chronically implanted 16-channel microwire array (Tucker-Davis Technologies, Alachua, FL; Figure 8B). The microwire array consisted of two rows of electrodes, with 8 electrodes per row. Multi-electrode stimulation was triggered in response to detected seizures while the rat moved around its cage. To accomplish this, a “line length” measure on each LFP channel, which has been shown to be effective for threshold based seizure detection, was calculated online (Esteller et al., 2001). A line length increment for a single LFP channel is defined as absolute difference between successive samples of the LFP,

(3) $$l_{k}\left[t\right]=|x_{k}\left[t\right]-x_{k}\left[t-T_{s}\right]|$$

where *x_k_*[*t*] is the LFP value on the *k*th channel at time *t*, and *T_s_* is the LFP sampling period of 500 μs. *l_k_*[*t*] was passed through a first order averaging filter,

(4) $$L_{k}^{\tau_{filt}}\left[t+T_{s}\right]=l_{k}\left[t\right]+\exp\left(\frac{-T_{s}}{\tau_{filt}}\right)\cdot \left(L_{k}^{\tau_{filt}}\left[t\right]-l_{k}\left[t\right]\right)$$

where *τ_filt_* is the filter time constant. For each recording channel, we calculated $L_{k}^{\tau_{filt}}\left[t+T_{s}\right]$ using two values of *τ_filt_*, 1 and 60 s, which resulted in short and long time averages that could be compared to detect rapidly occurring trends in *l_k_*[*t*]. Specifically, seizures were defined as events for which the criterion

(5) $$L_{k}^{1sec}\left[t\right]>2\cdot L_{k}^{60sec}\left[t\right]$$

was met on at least 4 of the 16 recordings channels. Upon seizure detection, 10 randomly chosen electrodes were stimulated sequentially at 45 Hz (aggregate frequency) for 10 s using biphasic, 1 V, 400 μs per phase, square waves. Figures 8C,D shows seizure events without and with closed-loop stimulation engaged. During stimulus application, $L_{k}^{\alpha }\left[t\right]$ values were frozen to prevent stimulation artifacts from affecting the line length averages.

There was no easily discernible effect of microstimulation on seizure duration or intensity during this pilot experiment. However, this proof of concept demonstrates the API’s utility in experiments conducted on freely moving animals and to modulate aberrant neural activity states. These features are useful for testing stimulation algorithms that do not just react to a seizure occurrence, but *predict* oncoming seizures ahead of time in order to apply a preventative action, which has proven a difficult goal to achieve (Mormann et al., 2007).

### 3.5 Silent barrage and robotic embodiment

The complexity of neural systems often necessitates intricate experimental protocols for proper investigation. To meet this requirement, the plugin API can be used to integrate NeuroRighter with complicated configurations external hardware and software. Working in collaboration with the SymbioticA group at the University of Western Australia, we used NeuroRighter for intercontinental neural control of a robotic system. This project was part of an art-science collaboration called Silent Barrage (Zeller-Townson et al., 2011), in which a dissociated cortical culture in Atlanta, Georgia, USA, was embodied with a remote array of robotic drawing machines situated in an interactive art gallery^9^. This system is an extension of the MEART project (Bakkum et al., 2007).

**Figure 9:**
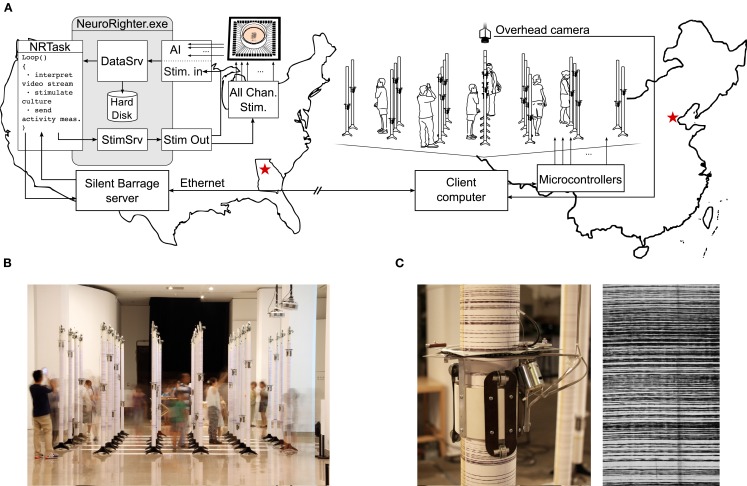
**The Silent Barrage robotic embodiment**. **(A)** Illustration of the Silent Barrage “organism” during its exhibition at the National Art Museum of China (NAMOC), in Beijing. Spatial patterns of action potentials recorded from a dissociated cortical culture are used to drive the robotic body. A video stream of visitors to the exhibition are interpreted by NeuroRighter’s plugin protocol and used to control multichannel electrical stimulation though the MEA, closing the loop around audience members, robotic system, and neural tissue over thousands of kilometers. **(B)** Audience members viewing the exhibition at NAMOC. Simultaneously, NeuroRighter translated the overhead video feed to stimulation patterns delivered to the culture and then translated resulting neuronal activity patterns to robotic actuation at the exhibit. **(C)** Photograph of an individual robot and the traces it produced during the NAMOC exhibition.

Figure 9A shows an illustration of the Silent Barrage system. Using the plugin API, a protocol was written to communicate between NeuroRighter and a custom web server running on the same computer. The web server in turn communicated with a client computer controlling a robotic body consisting of 32 independent robots. Each robot had a rotating actuator capable of climbing up and down a vertical column (Figure 9C). Columns were arranged in a grid that reflected the electrode layout of the MEA (Figures 9A,B). The height of each rotating actuator at a given moment was determined by the instantaneous firing rate detected on two adjacent electrodes from the 59-channel MEA. As the actuators traveled up and down, they periodically marked their positions on the vertical poles using an ink pen. Over time, this resulted in a visual record of spatiotemporal activity of the culture inscribed on each column (Figure 9C).

Silent Barrage was exhibited in the United States (New York), Spain (Madrid), Brazil (Sao Paolo), Ireland (Dublin), and China (Beijing). Visitors to the exhibitions were encouraged to mingle amongst the robotic embodiment and they were observed using overhead cameras (Figures 9A,B). The resulting video feed was processed on site to extract features of audience movement (Horn and Schunck, 1981) and these data were streamed back to NeuroRighter’s web server in Atlanta. Audience movement measures were then used to adjust stimulation patterns delivered through NeuroRighter’s all-channel stimulator. The relationship between incoming video data and electrical stimulation varied from exhibit to exhibit, from simple single-electrode rate coding schemes to more complex multi-electrode schemes where artificial neural networks were used to deliver certain stimulus pattern based upon learned features of incoming video data. Electrical stimulation modulated the activity state of the culture’s firing patterns, thus closing the loop around the dissociated culture, robotic body, and audience members separated by thousands of kilometers. While on exhibit in the National Art Museum of China, Silent Barrage was perhaps the Earth’s largest behaving “organism.”

## 4 Discussion

Closed-loop electrophysiology systems are powerful tools for neuroscience research because they can be used to parse recurrent systems into independently manipulable components. Voltage clamp techniques use feedback control to separate membrane potential from the recurrent influence of voltage-dependent ionic conductances (Marmont, 1949). Seminal experiments using voltage clamp have fostered our understanding of ion channels, neuronal excitability, and synaptic transmission. More recently, dynamic clamp has been used to deliver artificial transmembrane or synaptic conductances into living neurons (Prinz et al., 2004; Kispersky et al., 2011). Using these approaches, feedback control transforms dynamic features of *individual neurons* into controlled experimental variables. Similarly, closed-loop multichannel systems like NeuroRighter can transform features of *neural networks* into controlled experimental variables (Arsiero et al., 2007). NeuroRighter is a powerful tool for controlling network variables, improving upon currently available systems in terms of cost, usability, accessibility, extensibility, and hardware standardization (Wagenaar et al., 2006a; Stirman et al., 2011; Wallach et al., 2011; Ahrens et al., 2012). We have this demonstrated NeuroRighter’s power in conducting basic and translational neuroscience research through a variety of case studies.

Altered gene expression, synaptic input, or environmental conditions can induce changes in spiking activity, which in turn trigger activity-dependent processes. Because of this, it becomes difficult to distinguish the role these factors play in shaping network dynamics and neural plasticity independent of firing rate. Closed-loop multichannel feedback systems provide an opportunity to render the population firing rate a controlled experimental variable and enable study of cellular and network processes as a function of a defined activity state. We used Neurorighter to clamp the firing rate of a living neural network to user-defined setpoints over both short and long timescales (Sections 3.2, 3.3). Further, we were able to control population firing rate during prolonged application of the NMDA receptor antagonist, AP5 (Section 3.3). Our controller compensated for the loss of NMDA-mediated excitation and maintained network spiking at the target firing rate. Therefore, the effects of AP5 could be deduced through comparison with a control culture that underwent an identical clamping protocol but with intact synaptic transmission. In most studies that use long-term drug application, the individual roles of spiking and excitatory neurotransmission on plasticity are ambiguous (Turrigiano, 2011). By using a real-time multichannel feedback system, we have begun to unravel the independent effects of spiking and NMDAergic transmission on network behavior. This approach could also be used to more directly study the effects of altered genetic or environmental factors on network activity.

In addition to better controlled experimental variables, real-time feedback can be used to improve the relevance of experiments using reduced neural preparations in studies of behavior. Implicit to animal behavior is the interplay between motor output and sensory perception (e.g., head movement affects the visual input stream and vice-versa). While reduced neural preparations or immobilized animals provide excellent experimental accessibility, their major weakness is that they do not preserve a functional sensory-motor loop. We have demonstrated that Neurorighter is well-equipped for performing closed-loop experiments that restore the sensory-motor loop by interfacing living neural networks with artificial bodies (Section 3.5). The advantages of this approach over traditional open-loop techniques are twofold. First, neural systems can engage in “motor” behaviors without sacrificing delicate optical (Ahrens et al., 2012) or electrophysiological (Harvey et al., 2009) access due to actual motion. Secondly, the experimenter has complete control over the mapping between a recorded neural signal and its resulting “motor” effect (DeMarse et al., 2001; Ahrens et al., 2012). For example, Ahrens et al. (2012) recently examined optomotor adaptation in paralyzed larval zebrafish by embedding them in a virtual environment. Visual stimuli in the virtual environment provided a perception of motion, and induced fictive motor-nerve activity. Recorded motor-nerve activity was used to drive motion of the virtual environment. Changes in sensory-motor feedback gain could be achieved by adjusting the efficacy by which fictive motor patterns propelled the fish through its virtual world. All the while, full brain activity was recorded through single-cell resolution imaging, which would be nearly impossible to achieve in a freely moving animal. This study highlights how closed-loop interfaces between artificial bodies or environments and a living neural system allows excellent experimental access during behaviors requiring an intact sensory-motor loop.

Aside from basic research, closed-loop multichannel electrophysiology has possible medical applications. Predictive application of drugs or electrical stimulation has the potential to increase the efficacy and safety of treatments for various neurological disorders (Mormann et al., 2007; Rosin et al., 2011) and improve neural rehabilitation procedures (Jackson et al., 2006a). For example, a reliable seizure prediction algorithm would open the possibility for targeted interventions that abort seizures before they occur. Mormann et al. (2007) provide an extensive comparison of different methods for seizure prediction. Unfortunately, the clinical applicability of these algorithms remains quite pessimistic and future studies will require a high-throughput validation system to test robustness of seizure prediction algorithms under a variety of circumstances. We have demonstrated that NeuroRighter can be used for this purpose (Section 3.4). The stimulation algorithm we used is very similar to a method called responsive neurostimulation (NeuroPace Inc., Mountain View, CA, USA) that recently showed very promising results in a large, double-blind, pivotal clinical trial (Morrell, 2011). This form of closed-loop seizure modulation is not truly predictive as it was triggered on the occurrence of “unequivocal seizure onset” (Litt and Echauz, 2002). However, the API provides a means for easy reconfiguration in order to test alternative, predictive methods to abort seizures before they begin, using multichannel electrical stimulation or the local application of an anti-convulsive drug. Additionally, a plugin could be reconfigured for closed-loop modulation of other pathological neuronal activities or to facilitate motor rehabilitation (Jackson et al., 2006a).

Tools that enable closed-loop interaction with neural tissue at the network level have great potential to advance experimental neuroscience. Historically, open-source projects have been extremely good at adapting equipment and code designed for a singular purpose to other uses. For this reason, we envision a large role for open-source software and open-access hardware communities in the development of technologies for closed-loop eletrophysiology systems. Rapid improvements in microprocessor performance, embedded computer systems, on-chip multichannel signal processing, and A/D conversion technology must be matched by projects that can expose their powerful features for researchers with little or no background in embedded systems or computer science. NeuroRighter is one of several open-source hardware/software projects that are enabling more labs to carry out sophisticated electrophysiology with less money and more experimental flexibility^10^.

## Conflict of Interest Statement

The authors declare that the research was conducted in the absence of any commercial or financial relationships that could be construed as a potential conflict of interest.

## 5 Supplementary Material

The Supplementary Material for this article can be found online at http://www.frontiersin.org/Neural_Circuits/10.3389/fncir.2012.00098/abstract

## Footnotes

^1^https://sites.google.com/site/neurorighter/

^2^http://code.google.com/p/neurorighter/

^3^http://groups.google.com/group/neurorighter-users

^4^http://code.google.com/p/neurorighter/

^5^https://potterlab.gatech.edu/main/neurorighter-api-ref/

^6^http://www.mono-project.com/Main_Page

^7^http://code.google.com/p/neurorighter/source/browse/NR-ClosedLoop-Examples/

^8^http://www.arduino.cc/

^9^http://silentbarrage.com/

^10^http://www.its.caltech.edu/daw/meabench/ http://code.google.com/p/arte-ephys/ http://open-ephys.com/ http://www.backyardbrains.com/Home.aspx
